# Techniques of Deformity Correction in Adolescent Idiopathic Scoliosis—A Narrative Review of the Existing Literature

**DOI:** 10.3390/jcm14072396

**Published:** 2025-03-31

**Authors:** Aakash Jain, Kaustubh Ahuja, Simon B. Roberts, Athanasios I. Tsirikos

**Affiliations:** 1 All India Institute of Medical Sciences, Rishikesh 249201, India; akawadia7777@gmail.com; 2 Scottish National Spine Deformity Centre, Royal Hospital for Children and Young People, Edinburgh EH16 4TJ, UK; simon.roberts2@nhs.net

**Keywords:** adolescent idiopathic scoliosis, deformity surgery, surgical techniques

## Abstract

Surgical management of adolescent idiopathic scoliosis [AIS] is a complex undertaking with the primary goals to correct the deformity, maintain sagittal balance, preserve pulmonary function, maximize postoperative function, and improve or at least not harm the function of the lumbar spine. The evolution of surgical techniques for AIS has been remarkable, transitioning from rudimentary methods of spinal correction to highly refined, biomechanically sound procedures. Modern techniques incorporate advanced three-dimensional correction strategies, often leveraging pedicle screw constructs, which provide superior rotational control of the vertebral column. A number of surgical techniques have been described in the literature, each having its own pros and cons. This narrative review provides a detailed analysis of the contemporary surgical techniques used in the treatment of patients with AIS.

## 1. Introduction

Adolescent idiopathic scoliosis [AIS] is a complex three-dimensional deformity of the spine characterized by a coronal curvature of at least 10 degrees associated with vertebral rotation in the axial plane and sagittal imbalance, primarily presenting with thoracic hypokyphosis [1,2]. AIS is a common disease with a prevalence of 0.47–5.2% in the current literature [3]. AIS has significant implications for spinal alignment, pulmonary function, and psychosocial well-being [4,5]. Three fundamental treatment options exist in the management of this condition: observation, bracing, and scoliosis correction. Nearly 10% of AIS patients require some form of treatment and up to 0.1% will eventually require surgery [6].

Surgical management of AIS is a complex undertaking with a significant learning curve that extends many years into independent practice. The primary goals of surgery are to correct or improve the deformity, restore sagittal balance, preserve or improve pulmonary function, minimize morbidity or pain, maximize postoperative function, and improve or at least not to harm the function of the lumbar spine [7]. To accomplish these goals, surgical techniques may include anterior, posterior or combined anterior and posterior procedures. The evolution of surgical techniques for AIS has been remarkable, transitioning from basic methods of spinal correction to highly refined, biomechanically sound procedures. Historically, Harrington instrumentation represented a significant breakthrough by providing posterior distraction for coronal plane correction. However, its limitations in achieving sagittal alignment restoration and rotational correction led to the development of segmental instrumentation systems [8]. Modern techniques incorporate advanced three-dimensional correction strategies, often based on the use pedicle screw constructs, which provide superior rotational control and anchor stability compared to hooks and wires. These constructs, combined with strategic rod contouring and three-dimensional correction maneuvers, allow surgeons to address complex deformities with precision [9,10].

Additionally, anterior spinal fusion [ASF] techniques, once widely utilized for thoracic and lumbar AIS, have seen a decline with the advent of posterior-only approaches. However, ASF remains relevant in select cases due to its ability to reduce fusion levels and preserve motion segments. Emerging minimally invasive approaches, including thoracoscopic surgery, reflect ongoing innovation aimed at minimizing morbidity while maintaining surgical efficacy [11,12,13].

In parallel with advancements in instrumentation, the integration of intraoperative neuromonitoring [IONM], navigation, and imaging technologies has revolutionized AIS surgery. IONM enhances the safety profile of complex deformity corrections by providing real-time feedback on neural integrity [14]. Computer-assisted navigation and robotic-assisted systems are redefining precision in pedicle screw placement, further improving outcomes and reducing complications. Despite these advancements, AIS surgery remains a formidable undertaking, demanding meticulous planning and precise execution. Complications, including neurologic injury, implant failure, and pseudarthrosis, underscore the importance of comprehensive perioperative management. This review provides a detailed examination of contemporary surgical techniques for AIS.

## 2. Methodology

This review was conducted following established guidelines for systematic literature reviews. A comprehensive search was performed in the PubMed, Scopus, and Web of Science databases using the following keywords: “Adolescent idiopathic scoliosis”, “spinal fusion”, “deformity correction”, “Scoliosis techniques”, and “Surgical management.” Articles published between 2000 and 2024 were included, with a preference for high-quality studies, clinical trials, and systematic reviews. The inclusion criteria were studies reporting various techniques of deformity correction in AIS along with treatment outcomes and associated complications. Exclusion criteria included non-English articles, and studies without a description of the technique of deformity correction. Data extraction was performed independently by two authors, with disagreements resolved through discussion.

## 3. Preoperative Planning

### 3.1. Preoperative Clinical Evaluation in AIS

When surgery is considered for AIS, multiple factors influence decision making, including patient concerns, age, sex, pubertal status [Tanner stage], medical history, and prior treatments. A focused physical examination assesses key indicators such as shoulder and scapular asymmetry, trunk rotation [Adam’s forward bend test with the use of a scoliometer], curve flexibility, sagittal and coronal balance, pelvic obliquity, leg length discrepancy, and neurological abnormalities. Clinical photographs from various angles further aid surgical planning.

The Lenke classification [7] is the most widely used method for categorizing AIS. It was developed to provide an approach to classify spinal deformities based on coronal, sagittal, and structural characteristics. The classification aids in treatment planning, particularly in deciding the levels for spinal fusion. It helps spine surgeons tailor surgical strategies to optimize spinal correction while maintaining balance [Table 1].

### 3.2. Perioperative Respiratory Care in AIS

Perioperative respiratory care plays a crucial role in the management of patients with AIS, particularly those with significant thoracic deformities that impact pulmonary function. Preoperative respiratory assessment is essential to identify and optimize patients at risk of restrictive or obstructive lung disease. Severe thoracic scoliosis with preserved kyphosis is often associated with restrictive lung disease due to reduced thoracic cage compliance and diminished lung volumes. In contrast, patients with thoracic scoliosis and a lordotic thoracic spine may develop obstructive lung disease as spinal penetration into the chest narrows the posteroanterior chest diameter, leading to direct airway compression and impaired pulmonary mechanics [15,16].

## 4. Surgical Correction

Surgery is required in approximately 10% of patients with AIS, typically when Cobb angles exceed 40–45° in skeletally immature patients or progress beyond 50° at the end of spinal growth [17]. Proper selection of fusion levels is crucial, as poor choices may lead to spinal imbalance, junctional kyphosis and the need for revision surgery. At the same time, unnecessary extension of the fusion beyond the required vertebral levels can limit spinal mobility and accelerate adjacent segment degeneration [18].

The principle of selective fusion aims to preserve motion segments while maintaining global spinal balance. Some thoracic curves correct spontaneously after a selective thoracolumbar fusion, and preserving at least three unfused lumbar segments is associated with better long-term outcomes and reduced risk of back pain. However, there is no universally accepted method for determining the lowest instrumented vertebra [LIV]. In Lenke Type 1 and 2 curves with a lumbar C modifier, the lumbar curve may be included if there is significant truncal translation, waistline asymmetry, and/or apical vertebral rotation. For lumbar A or B modifiers, the “touched vertebra” rule is applied, where the LIV is the most cephalad vertebra intersected by the central sacral vertical line [CSVL]. In Lenke Type 3–6 curves, the lumbar curve is typically fused, with the distal lumbar end vertebra serving as the caudal level. Scoliosis flexibility plays a significant role when deciding the LIV with curves that retain flexibility, as evident on supine traction or fulcrum bending radiographs, allowing the caudal level of the fusion to be one level proximal to the distal end vertebra, with the aim to maintain three mobile segments in the lumbar spine and stop the fusion at L3 instead of L4.

Upper instrumented vertebra [UIV] selection focuses on shoulder balance and minimizing the development of proximal junctional kyphosis. If the proximal thoracic curve requires fusion, the construct may extend to T2 or T3; otherwise, fusion may start at T4 or T5. If the shoulder imbalance and T1 tilt align in the same direction, a full fusion of the proximal thoracic curve is recommended. When the opposite is true, selective fusion of the main thoracic curve may suffice. The clavicle angle helps determine the need for proximal thoracic fusion—an elevated left shoulder or significant T1 tilt [>5°] may warrant fusion of the entire proximal thoracic curve. In a practical way, the position of the shoulders can help determine the need for proximal extension of the fusion in the presence of a double left upper thoracic and right mid thoracic scoliosis. If the left shoulder is higher in the standing scoliosis X-ray, the UIV can be T2, if the shoulders are level, this can be T3, and if the right shoulder is elevated, the upper end of the fusion can stop at T4.

Sagittal alignment is critical for long-term outcomes and quality of life. Spinopelvic parameters such as thoracic kyphosis, lumbar lordosis, pelvic incidence, pelvic tilt, and sacral slope should be assessed preoperatively. Surgical goals include achieving lumbar lordosis within 10° of pelvic incidence to maintain global sagittal balance. Careful preoperative planning incorporating these considerations optimizes outcomes and reduces complications.

## 5. Factors Influencing Deformity Correction

The degree of deformity correction achieved during posterior spinal instrumentation and fusion [PSIF] is influenced by several key factors. One critical determinant is the inherent flexibility of the spine, which varies based on the patient’s age, curve characteristics, and underlying spinal biomechanics. Another significant factor is the magnitude and direction of corrective forces applied during surgery, including distraction, compression, derotation, and translation forces. The surgeon is pivotal in optimizing these factors by selecting appropriate preoperative strategies, such as curve assessment through flexibility radiographs [fulcrum bending] or supine traction imaging, and executing precise intraoperative techniques. Additionally, the choice of instrumentation, including pedicle screws, rods, and advanced tools like shape memory alloys, further enhances the ability to achieve effective and balanced deformity correction.

### 5.1. Adequate Spinal Release

Performing spinal release, with the aim to increase flexibility of the vertebral column across the levels of the scoliotic deformity that will allow optimum curve correction, can be performed using posterior or anterior techniques.

### 5.2. Posterior-Based Osteotomies

**Figure 1:**
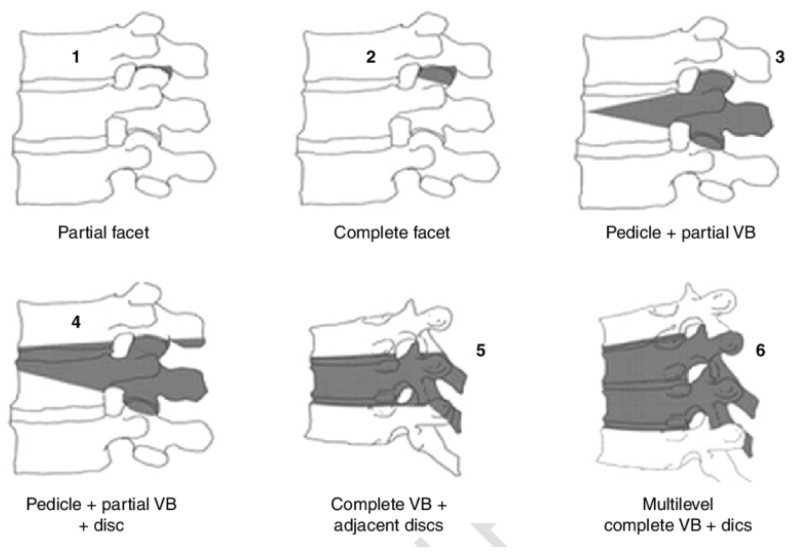
Schwab anatomical spinal osteotomy classification [19].

Osteotomies are valuable surgical techniques employed to improve spinal mobility and facilitate deformity correction in AIS. Schwab et al. introduced a comprehensive classification system for spinal osteotomies, categorizing them into six grades based on the extent of anatomical resection [Figure 1]. This classification ranges from grade 1, which involves the partial resection of the facet joints, to grade 6, which entails the resection of multiple vertebrae and intervertebral discs [19]. Grade 1 and 2 osteotomies are applicable in the treatment of AIS.

- Grade 1 osteotomies are routinely utilized in posterior spinal instrumentation and fusion [PSIF] for AIS. These osteotomies enhance the visualization of anatomical landmarks for pedicle screw placement, provide autograft material, increase spinal mobility, and facilitate interfacetal fusion.

- Grade 2 osteotomies, which involve a more extensive resection of the posterior column, are often employed to address specific deformity characteristics. While traditionally used for coronal plane correction, their primary benefit lies in improving sagittal plane mobility, particularly in cases of hypokyphosis or hyperkyphosis. Shah et al. conducted a study involving 87 consecutive AIS patients who underwent posterior spinal fusion with pedicle screw constructs and grade 2 osteotomies. In patients with preoperative hypokyphosis [T5–T12 < 20°], kyphosis improved significantly from 8.1° to 18.3° [*p* < 0.001]. For those with hyperkyphotic deformities [T5–T12 > 40°], kyphosis was effectively reduced from 45° to 26° [*p* < 0.001] [20]. Similarly, Samdani et al. analyzed 199 AIS patients with Lenke 1A and 1B curve types. Among these, 125 patients underwent an average of 4.3 levels of grade 2 osteotomies. Compared to those treated with grade 1 osteotomies, the grade 2 group achieved superior outcomes, including greater thoracic Cobb angle correction [67.1% versus 61.8%, *p* = 0.01], enhanced improvement in T5–T12 kyphosis [13.0° versus 20.4°, *p* = 0.045], and better rib prominence correction [53.2% versus 38.4%, *p* = 0.02] [21]. Wang et al. further evaluated 80 consecutive Lenke type 1 AIS patients with hypokyphotic curves who underwent posterior spinal fusion. Patients were divided into two groups based on whether Ponte osteotomies were performed. Both groups demonstrated postoperative improvements in sagittal and coronal alignment, but the Ponte group showed significantly better outcomes. At the two-year follow-up, the main thoracic [MT] angle was reduced more effectively in the Ponte group [15.18° ± 2.84° versus 20.33° ± 3.75°, *p* < 0.001], and thoracic kyphosis [TK] was better restored [24.23° ± 2.71° versus 19.93° ± 2.38°, *p* < 0.001]. These findings highlight the utility of Ponte osteotomies in achieving superior coronal correction and sagittal contour restoration in AIS patients with hypokyphosis [22]. Despite their benefits, grade 2 osteotomies are associated with increased surgical complexity. Halanski and Cassidy reviewed 37 patients undergoing PSIF for AIS [Lenke types I and II] and reported higher estimated blood loss per level [97 ± 42 mL versus 66 ± 25 mL, *p* = 0.01] and longer operative time per level [31 ± 5 min versus 23 ± 3 min, *p* < 0.001] compared to grade 1 osteotomies. In contrast to previous reports, no significant improvement in curve correction was observed with the use of grade 2 osteotomies in this study [23].

Faldini et al. conducted a systematic review and meta-analysis to evaluate the efficacy and safety of Ponte osteotomies [POs] in AIS surgery. Analyzing nine studies, they found that POs significantly improved thoracic kyphosis [TK] in hypokyphotic patients [+6.6°, *p* < 0.01] but did not yield significant changes in normokyphotic patients [+0.2°, *p* = 0.96] or provide superior coronal correction. The use of POs was associated with increased estimated blood loss and longer surgical time, with a trend toward higher complication rates [24]. There is no universal consensus on the indications for specific types or frequencies of osteotomy use in AIS. Grade 2 osteotomies are typically reserved for patients with thoracic sagittal plane deformities [e.g., hypokyphosis < 10° or hyperkyphosis > 40°] or severe, rigid coronal deformities [>70–90°] with limited flexibility [>40° on dynamic imaging]. By tailoring osteotomy selection to the patient’s unique spinal deformity and mobility, surgeons can achieve optimal outcomes while balancing risks and benefits.

### 5.3. Anterior Release

Anterior release is an alternative method to improve spinal mobility, historically utilized for managing large, stiff curves or thoracic hypokyphosis. However, its popularity has declined with the advent of segmental pedicle screw instrumentation that can achieve better coronal curve correction compared to older techniques. Despite this, it remains a valuable option for spinal deformity surgeons, particularly for severe, rigid curves [>90° with <25% correction on bending radiographs] or deformities accompanied by significant thoracic lordosis [>10°] [25]. The procedure can be performed via open or thoracoscopic approaches, both of which carry potential risks of pulmonary function impairment due to chest wall disruption.

A systematic review by Lee et al. of 22 studies found that anterior release followed by posterior spinal instrumentation and fusion [PSIF] results in moderate pulmonary function improvement at two years postoperatively. In contrast, anterior spinal fusion [open or thoracoscopic] causes a temporary decline in pulmonary function, which normalizes to preoperative levels within two years [26]. Sucato and Elerson highlighted that thoracoscopic release performed in the prone position offers similar safety and efficacy to the lateral position while avoiding single-lung ventilation and patient repositioning, with no detrimental effect on pulmonary function [27,28]. A six-year prospective study evaluating the outcome of combined anterior and posterior spinal fusion [A/PSF] in AIS found no long-term pulmonary decline, with FEV1 and FVC returning to preoperative levels. Quality of life, assessed via SRS-22, showed significant improvement, with high patient satisfaction [4.8/5], reinforcing the effectiveness of A/PSF in correcting scoliosis while preserving lung function [29].

An additional advantage of anterior release for thoracic AIS is that it allows performing an anterior thoracoplasty across the levels of the scoliotic deformity which achieves predictable improvement in the cosmetic aspect of convex rib prominence by excising the rib heads and adjacent 3–4 cm of ribs. This creates a “flail” convex hemithorax that heals in an improved position once the scoliosis has been corrected and the spine has fused, resulting in high patient satisfaction [29].

Moreover, the addition of an anterior spinal release to the posterior instrumented fusion can achieve a circumferential 360° fusion that prevents the risk of crankshaft effect in younger patients due to remaining anterior vertebral body growth. There is, however, an argument that modern segmental pedicle screw constructs which provide three-column spinal fixation and increased rigidity can prevent the development of the crankshaft phenomenon, obviating the need for an additional anterior spinal fusion.

Anterior release of the spine as part of deformity correction necessitates ligation of segmental vessels across several segments along the length of the curvature. Disrupting the spinal cord’s blood supply can potentially lead to permanent neurological compromise, with reported risk rates ranging from 0% to 0.86%. Tsirikos et al. conducted a retrospective review of 346 pediatric and adolescent patients who underwent anterior spinal deformity surgery with segmental vessel ligation; 2651 segmental vessels were ligated, and intraoperative spinal cord monitoring was used in 331 cases. Only one neurological complication was observed in a patient with congenital scoliosis and complex intraspinal anomalies. This indicates that unilateral segmental vessel ligation is generally safe, except in cases involving complex congenital spinal deformities with associated vascular abnormalities [30].

Even though anterior release is expected to aid in restoring thoracic kyphosis, clinical evidence does not consistently support this. Ferrero et al. studied 56 AIS patients with reduced kyphosis [T5–T12 < 20°] and found no significant difference in thoracic kyphosis restoration between those undergoing staged thoracoscopic release and posterior-only approaches [18.3° ± 13.6° versus 15.2° ± 9.0°, *p* = 0.35] [31]. Similarly, Shi et al. compared patients treated with a combined thoracoscopic anterior release and PSIF [using hybrid fixation] versus PSIF alone [with all-pedicle screw constructs] and found no significant differences in coronal or sagittal plane correction [12]. While anterior release may benefit specific deformities, its indications should be carefully considered given the lack of consistent evidence for superiority over posterior-only approaches.

### 5.4. Rod Factors

Rod stiffness, a fundamental biomechanical property, refers to the rod’s resistance to deformation under applied loads. It plays a pivotal role in determining the corrective power of a construct. Rod stiffness is influenced by two primary factors: the inherent modulus of elasticity of the material and the rod’s diameter. For a cylindrical structure like a spinal rod, bending rigidity is proportional to the radius raised to the fourth power, meaning even a slight increase in diameter significantly enhances stiffness. However, increased stiffness is not universally beneficial. Excessive rod stiffness can surpass the strength of the bone–implant interface, potentially causing implant pullout or catastrophic failure. To optimize outcomes, surgeons must balance rod stiffness with the deformity’s characteristics and the quality of spinal anchor points. This tailored approach ensures sufficient correction without compromising the integrity of the construct or the patient’s bone–implant interface. Research highlights that larger rod diameters do not always guarantee better outcomes. Prince et al., in their review of 352 AIS patients treated with posterior spinal instrumentation and fusion [PSIF], compared 5.5 mm and 6.35 mm rods. They found no significant difference in coronal or sagittal plane correction between the two groups, indicating that rod size alone may not directly correlate with improved clinical outcomes.

The choice of rod material is another critical consideration. Commonly used materials include stainless steel [SS], cobalt–chromium [CoCr], and titanium [Ti] alloys, each with unique biomechanical and clinical properties:

- Stainless steel [SS]: With the highest modulus of elasticity among the three, SS rods provide superior stiffness and resistance to deformation, making them ideal for complex deformity corrections. However, their high stiffness can pose challenges in osteopenic patients due to increased stress at the anchor points.
- Cobalt–chromium [CoCr]: CoCr rods offer intermediate stiffness, providing a balance between rigidity and flexibility. They are also effective for deformity correction but less prone to extreme stiffness-related complications compared to SS. CoCr rods can provide superior frontal plane correction with higher corrective forces and minimal deformation compared to SS rods [32].
- Titanium [Ti]: Ti rods, with the lowest modulus of elasticity, are more elastic and forgiving, making them suitable for patients with poorer bone quality. Ti rods are less prone to corrosion, produce fewer imaging artifacts, and may reduce the risk of bacterial glycocalyx formation, potentially lowering surgical site infection [SSI] rates. However, their flexibility limits their utility in precise deformity corrections requiring in situ contouring. The use of higher diameter Ti rods increases their stiffness and may be the “happy medium” for deformity correction in AIS.

Bowden et al. evaluated the impact of different rod materials and diameters on surgical outcomes in adolescent idiopathic scoliosis [AIS] surgery. They found that cobalt–chromium [CoCr] rods provided significantly better thoracic kyphosis correction than titanium [Ti] rods, both at short-term follow-up [0–3 months, MD = −2.98°, *p* = 0.04] and long-term follow-up [≥24 months, MD = −3.99°, *p* = 0.009]. Additionally, 5.5 mm rods were associated with lower reoperation rates [1%, 95% CI 0.0–3.0%] compared to 6 mm rods [6%, 95% CI 2.0–9.0%, *p* = 0.04]. However, no significant differences were observed in coronal angle correction, lumbar lordosis, proximal junctional kyphosis, revision or infection rates across rods of different materials or diameters [33]. The debate over SSI rates between SS and Ti rods remains unresolved. Some studies suggest Ti rods may lower infection risks, while others find no significant difference.

### 5.5. Pedicle Screw Factors

Pedicle screws are essential for achieving strong fixation to the spine and stable instrumentation constructs, and their effectiveness depends on several factors, including core geometry, thread design, material properties, and insertion technique. Modern pedicle screws come in three primary designs:

- Monoaxial screws: These have a fixed head, providing the highest rigidity and stability. They are ideal for deformity correction requiring precise control, such as direct vertebral rotation [DVR]. However, they necessitate exact rod contouring to fit the saddle, which increases stress at the bone–screw interface, raising the risk of fixation failure.
- Uniaxial screws: These screws allow motion in a single plane, typically the sagittal plane, while maintaining rigidity in other directions. They strike a balance between rigidity and ease of rod insertion, making them suitable for axial deformity correction.
- Polyaxial screws: Featuring a spherical joint between the screw head and shaft, polyaxial screws allow multi-directional movement, simplifying rod placement and accommodating coronal plane malalignment. While user-friendly, they provide less vertebral derotational forces compared to monoaxial or uniplanar screws.

Studies support the nuanced use of these designs based on deformity characteristics. Kuklo et al. compared monoaxial and polyaxial screws in AIS patients and found superior axial plane correction with monoaxial screws [34]. Similarly, Dalal et al. demonstrated that uniplanar screws provided improved apical vertebral rotation compared to polyaxial screws, making them preferable in regions where axial control is crucial [35]. However, Yang et al., claimed that the use of polyaxial pedicle screws resulted in coronal, sagittal, and rotational correction outcomes comparable to those associated with the use of monoaxial pedicle screws for surgical treatment using PSI and RD to treat moderate cases of AIS [36].

Anchor density, defined as the number of spinal anchors per fusion level, directly influences the corrective power of a spinal construct. Higher anchor density provides better fixation and minimizes rod deformation, particularly in rigid or large curves. However, increased density comes with trade-offs, including prolonged operative time, higher costs, and potential complications. As per the current literature, it is still unclear whether screw density results in better clinical and radiographic outcomes in AIS patients [37,38,39,40,41]. Qiang et al. conducted a systematic review and meta-analysis of 11 studies involving 697 patients and found that low-density pedicle screws can achieve similar clinical and radiographic outcomes compared with high-density constructs in patients with Lenke I AIS while potentially reducing costs, making it a more cost-effective option without compromising patient outcomes [41]. In contrast, Larsen et al. conducted a retrospective review of 952 patients with Lenke type 1, 2, or 5 curves revealing better radiographic and patient-reported outcomes in high-density constructs [>1.54 anchors per level] compared to low-density constructs [40].

### 5.6. Correction Techniques

Deformity correction in the treatment of AIS can be achieved with the use of anterior or posterior techniques. Posterior techniques can be divided into dual-rod and single-rod correction. Single-rod correction can be further divided into concave-sided or convex-sided correction. The maneuvers usually used for scoliosis correction include rod derotation, cantilever correction, segmental vertebral derotation, en bloc vertebral derotation, compression–distraction forces and in-situ rod bending.

### 5.7. Biomechanics of Corrections Maneuvers

Correction in scoliosis deformity involves changes in both the soft tissue and bone tissue planes. At a microscopic level, the material properties of specific bony structures, such as lamellae, and soft tissues vary widely, including differences in elastic modulus and contact hardness. When analyzing the stress–strain curve of compact cortical bone, the interval between the yield point and the fracture point is too narrow to achieve significant deformation without causing fractures. Thus, bony tissue corrections necessitate bony resection such as when performing osteotomies, as the changes in bone during corrective maneuvers are minimal and not observable.

In contrast, the viscoelastic properties of soft tissues allow for substantial deformity correction without compromising tissue integrity. Viscoelasticity, the characteristic of materials exhibiting both viscous and elastic behavior under deformation, plays a critical role in this process. Elasticity arises from bond stretching within crystalline solids, while viscosity results from molecular diffusion in amorphous materials. Viscoelastic materials, such as tendons and ligaments, exhibit creep—molecular rearrangement under stress—allowing them to sustain deformation and maintain the new shape unless subjected to counteracting forces. This property makes time a crucial factor in corrective maneuvers, as prolonged application of stress enhances deformity correction [Table 2].

### 5.8. Global Rod Derotation

**Figure 2:**
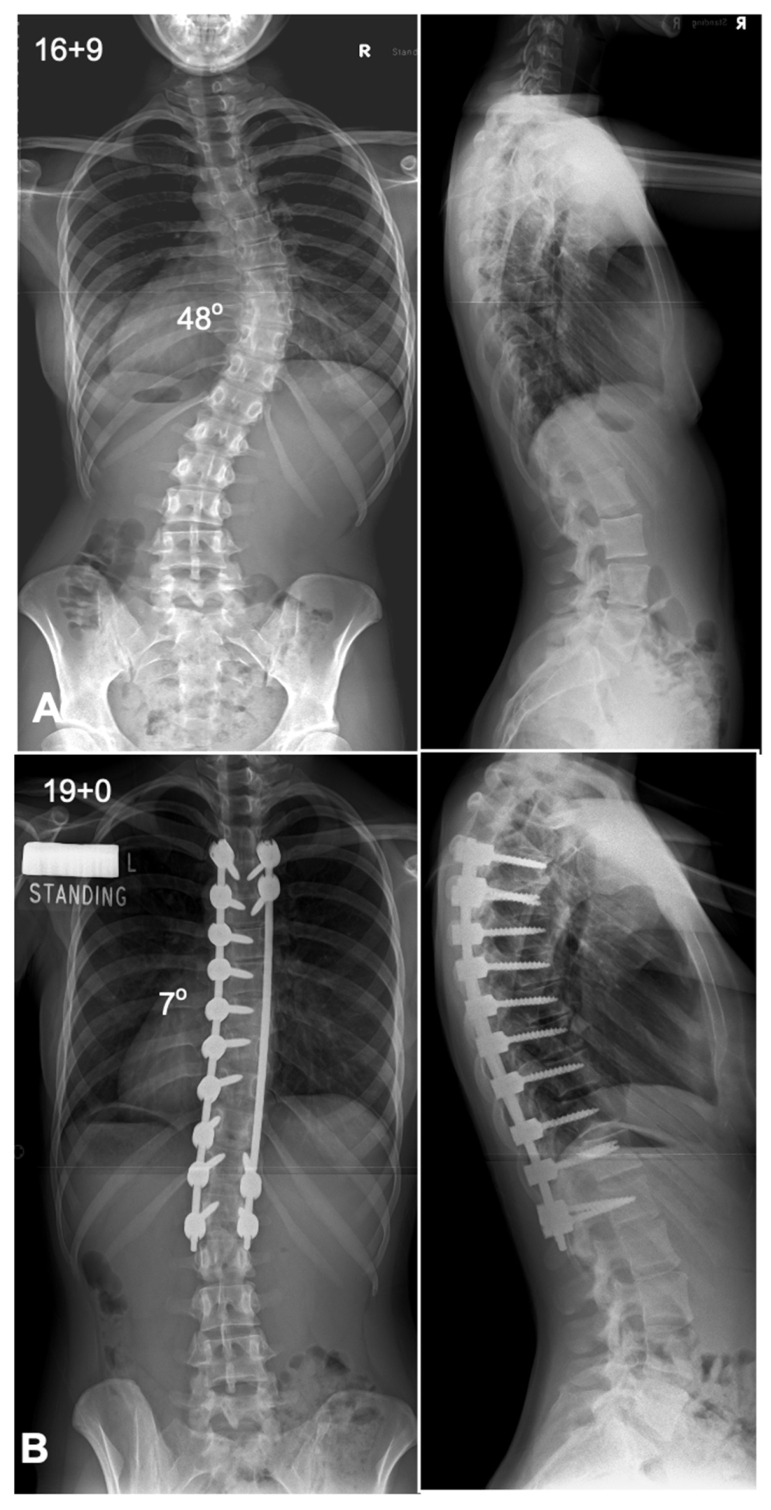
Deformity correction using a typical concave side rod derotation maneuver. (**A**) Preoperative antero-posterior and lateral radiographs showing a thoracic scoliosis with a Cobb angle of 48 degrees. (**B**) Postoperative radiographs showing good deformity correction with coronal and sagittal balance.

The global rod derotation technique, originally introduced by Cotrel and Dubousset, is based on the concept that the coronal deformity typical in AIS approximates the desired sagittal alignment when rotated 90° [51]. This method involves inserting a rod pre-contoured to the ideal sagittal shape on the curve’s concavity. The anchors are loosely attached to the rod and then rotated 90° along the axial plane to transform the coronal deformity into sagittal alignment [52,53]. The traditional use of the concave side for scoliosis correction poses challenges due to anatomical constraints. Studies, including that by Liljenqvist et al., reveal significantly narrower pedicles on the concave side at the thoracic curve apex, increasing the risk of cortical wall penetration [54]. Anekstein et al. evaluated the use of a convex rod derotation technique for correcting AIS in 40 patients treated with all-pedicle screw constructs and followed for two years. The mean preoperative Cobb angle of 60° improved to 17° postoperatively, with a 7% correction loss at follow-up, yielding a 71% major curve correction. The technique showed comparable outcomes to traditional concave-side corrections, with additional benefits of reduced neurological risks due to safer screw placement on the convex side. Thoracic kyphosis decreased slightly, and no major complications, infections, or revisions were reported [55]. This approach benefits from the stability offered by multiple fixation points, distributing mechanical stress and reducing localized anchor strain. It is particularly effective for thoracic hypokyphosis, enabling simultaneous correction in both the coronal and sagittal planes. However, this technique does not correct axial plane deformities. Advanced imaging has shown that while previously thought to induce rotational correction at the apex, it primarily results in translational adjustment. Additionally, the ideal sagittal contour may not align with the coronal deformity, potentially leading to suboptimal sagittal apex placement post-rotation [46,56]. A case example is shown in Figure 2.

### 5.9. Mechanism of Curve Correction by Rod Derotation Maneuver

In correcting a thoracic curve, a contoured rod is first positioned on the concave side following appropriate soft tissue and bony releases. Plugs are placed at the anchor points [between the screw or hook and the rod], but are not initially tightened. The rod is then rotated toward the concavity, generating a strong postero-medial traction force on the apical and juxta-apical vertebrae. Instead of simply rotating, these vertebrae translate toward the midline and move posteriorly. Theoretically, this maneuver also facilitates some degree of apical vertebral derotation in the transverse plane. However, for this rotational correction to be effective, minimal friction must exist at the screw–rod interface, allowing free movement of the screw along the rod. This optimal scenario is primarily achievable in smaller, less rigid curves. In most cases, friction at these junctions is significant and may paradoxically increase rotational deformity, thereby exacerbating the rib hump. Clinically, the impact of rod derotation on rotational correction in scoliosis is minimal.

Despite this, rod rotation provides substantial coronal and sagittal plane correction. The sagittal realignment results from the movement of the upper and lower instrumented vertebrae during the 90° rod rotation. The upper instrumented vertebra shifts anteriorly and flexes, while the lower instrumented vertebra also moves anteriorly but extends in the sagittal plane. These adjustments help restore the sagittal profile during correction. Additionally, the rotational and translational effects extend beyond the instrumented segments, influencing adjacent spinal regions. This is particularly relevant in selective thoracic fusion for major thoracic-compensatory lumbar curves, where excessive derotation of the primary thoracic curve can negatively impact the spontaneous correction of the compensatory lumbar curve.

When comparing all-pedicle screw [AS] and hybrid hook–screw [HS] instrumentation in the treatment of AIS, Tsirikos and McMillan found that both achieved comparable scoliosis correction [≥75%], but the HS group provided better restoration of thoracic kyphosis and global sagittal balance while reducing surgical time, blood loss, and implant cost. These findings align with the biomechanical effects of rod rotation, as excessive implant density in AS constructs flattens the rod on the concave side and may limit sagittal restoration. Additionally, both techniques demonstrated low complication rates, with no neurological or visceral injuries, supporting the HS construct as a viable alternative for three-dimensional deformity correction [57].

In the lumbar spine, the rod is applied to the convex side of the curve and rotated clockwise [as viewed from the caudal end] to restore lumbar lordosis. Unlike in the thoracic spine, rod rotation in the lumbar region does not worsen apical rotational deformity but instead reduces it, as the direction of rotation is opposite to that used in thoracic correction. Here, the spinous processes, which are deviated toward the concavity, are pulled back toward the midline as lumbar scoliosis is converted into lumbar lordosis. Consequently, friction at the rod–tulip interface is less of a concern, as it aids in derotating the apical and juxta-apical vertebrae.

A critical principle in rod rotation is that the rod should always be rotated toward the side where it is applied—toward the concavity in the thoracic spine and the convexity in the lumbar spine. This approach is crucial because the medial wall of the pedicle is approximately three times stronger than the lateral wall. If a rod is applied on the convex side of a thoracic curve and rotated in the same direction, it exerts a posteromedial pull at the apex, increasing the risk of pedicle screw breach through the weaker lateral wall.

### 5.10. Simultaneous Double-Rod Rotation

Ito and colleagues introduced a modified rod rotation technique to address the limitations of traditional single-rod rotation [58]. In the standard method, the concave rod in a thoracic curve often flattens during rotation, particularly with modern titanium rods, which are more flexible than the previously used stainless steel rods. This flattening can compromise the sagittal profile, leading to suboptimal clinical outcomes.

**Figure 3:**
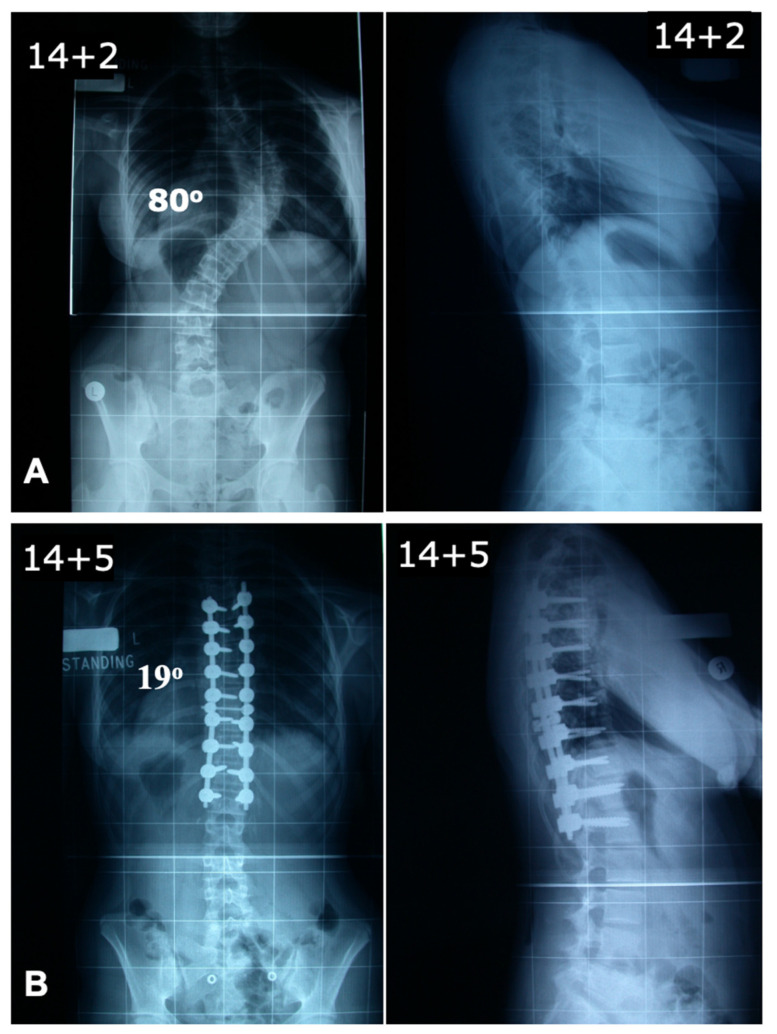
Deformity correction using double-rod technique. (**A**) Preoperative antero-posterior and lateral radiographs showing a thoracic scoliosis with a Cobb angle of 80 degrees. (**B**) Postoperative radiographs showing good deformity correction with coronal and sagittal balance.

In contrast, the simultaneous double-rod rotation technique involves inserting rods on both sides of the curve and rotating them together (Figure 3). This synchronized movement corrects both the coronal and sagittal profiles while preventing the flattening of either rod. Additionally, this approach enhances rotational deformity correction. The concave rod in a typical thoracic curve is bent more acutely than the convex rod, requiring it to travel a greater distance during rotation. This differential movement generates a rotational moment at the apical vertebra, promoting its derotation. The primary corrective force applied in this technique is a synchronized, posteromedial upward push on the spine.

### 5.11. Vertebral Translation

In the translation maneuver, the technique obtains most of its reduction by gradually translating the periapical vertebrae toward the rod in the midline. This technique repositions spinal segments toward a pre-contoured rod using a range of anchors, including standard pedicle screws, reduction screws, or sublaminar devices, namely wires or tapes [53]. The process involves attaching the rod proximally and distally, then gradually drawing intermediate anchors and vertebrae toward the rod with specialized tools. The rod is secured proximally and distally before fine-tuning the apical translation, allowing for viscoelastic adaptation during gradual correction. This method facilitates thoracic kyphosis restoration when the vertebrae are translated posteriorly to the rod. However, it demands secure fixation, making it less suitable for poor bone quality. Care should be taken to look for evidence of anchor pullout during the reduction maneuvers.

### 5.12. Cantilever Maneuver

In this surgical correction technique, six groups of pedicle screws are inserted into the upper, apical, and lower segments on both sides of the curve. Once the screws are in place, a pre-bent rod is secured to the pedicle screws on the convex side. Two long in situ benders are then attached above and below the apical pedicle screws on the convex side, acting as lever arms in the coronal plane. By bringing the free ends of these lever arms closer together, a strong corrective force is generated to realign the curve in the coronal plane.

If additional correction is required in the sagittal plane, another pair of in situ benders is secured to the rod above and below the apical pedicle screws. These function as lever arms in the sagittal plane, applying corrective forces through the cantilever bending technique. A second pre-bent rod, shaped to match the corrected alignment, is then fixed to the screws on the concave side to stabilize and maintain the achieved correction.

Once both rods are connected with transverse links, fine adjustments are made to the end vertebrae using intraoperative PA radiographs to ensure proper spinal balance. Finally, the lever arms are released, completing the correction.

Chang et al. found the cantilever bending technique alone [without the use of anterior release] as an effective technique for the correction of large and rigid scoliotic deformities of any etiology [59]. Applying reduction forces to multiple screws simultaneously and extending the patient’s trunk by adjusting the operating table can prevent screw pull-out. This technique is advantageous in managing hyperkyphotic deformities by enabling controlled lordosis induction [60].

### 5.13. Vertebral Coplanar Alignment

Vertebral coplanar alignment [VCA] is an advanced technique introduced by Vallespir et al. for standardized three-dimensional correction in scoliosis surgery [53]. Unlike traditional methods that rely on rod manipulation to achieve correction, VCA realigns the vertebral axes before rod placement, while reducing mechanical stress on instrumentation and improving precision. In a normal standing spine, the anteroposterior [*X*-axis] and transverse [*Z*-axis] of the vertebrae are coplanar, but scoliosis disrupts this alignment due to rotational and translational deformities across all three planes. VCA restores this coplanarity while also correcting thoracic hypokyphosis by re-establishing the posterior divergence of the *X*-axis. The procedure begins with the insertion of monoaxial pedicle screws on the convex side of the curve, followed by the attachment of slotted stainless steel tubes aligned with the anteroposterior axis of each vertebra. A rigid rod is then inserted through these slots, gradually bringing the vertebrae into a single rotational axis. Polyethylene spacers are placed between the tips of the slotted tubes to restore kyphosis, and a second rod is inserted beneath the first, further driving the vertebrae into coplanar alignment. Once the correction is achieved, a definitive rod is secured on the concave side before the slotted tubes and convex rod are removed. VCA offers advantages over conventional techniques, including true three-dimensional correction, minimized stress on instrumentation, improved neurological safety by reducing the risk of pedicle screw breach, and better load distribution across the instrumented segments. Additionally, by achieving correction before rod placement, the technique simplifies rod insertion and reduces implant-related complications. A clinical study on 25 patients with Lenke Type 1 adolescent idiopathic scoliosis demonstrated good outcomes, with an average of 73% coronal correction in thoracic curves, 70% correction in thoracolumbar curves, 56% apical vertebral derotation, and 65% rib hump reduction without the need for thoracoplasty. Importantly, thoracic kyphosis was preserved, preventing excessive flattening often seen with other techniques [61].

### 5.14. Vertebral Derotation

Pedicle screws enable three-column fixation, allowing for precise corrective forces to be applied in axial plane correction. In contrast, other types of anchors are limited in this regard, as they attach posteriorly at the vertebra’s instantaneous axis of rotation [IAR] in the axial plane. One of the most prominent cosmetic concerns in AIS is the rib hump deformity caused by vertebral rotation. Over time, various surgical techniques have evolved to address this deformity, including segmental derotation and en bloc derotation. Both methods aim to correct spinal rotation and enhance cosmetic outcomes by reducing rib prominence.

Lee et al. originally described direct vertebral rotation using segmental pedicle screw fixation, demonstrating superior rotational and coronal correction compared to simple rod derotation. This technique can be performed in various ways. In this method, after or during concave rod rotation, screw derotators—typically monoaxial—are inserted into the juxta-apical screws on both the concave and convex sides and rotated in the opposite direction of the rod [43]. In contrast, the vertebral coplanar alignment [VCA] technique, proposed by Vallespir et al., utilizes slotted tubes attached to monoaxial screws at each level on the convex side [61]. Qiu et al. found that the VCA technique achieved comparable coronal correction to derotation in Lenke 1 scoliosis but provided superior thoracic kyphosis restoration. Both techniques had similar safety profiles, though screw pullouts and hemothorax occurred in the derotation group [62]. Another widely adopted approach, vertebral column manipulation, introduced by Chang and Lenke, involves derotation from either the convex or concave side. This technique initially uses a derotator device to “triangulate” periapical pedicle screws, which are then connected into a “quadrilateral” frame for enhanced correction [59].

Before initiating these corrective maneuvers, it is crucial to identify and secure the rostral and caudal neutral vertebrae using derotation devices, which serve as counterforces during rotational adjustments. These devices or tubes are then attached to adjacent screws to apply corrective axial forces. The convex derotation device is directed downward and medially, while the concave device is pulled upward. Simultaneously, the neutral vertebrae are shifted toward the opposite side, with additional downward forces applied over the rib hump to enhance correction.

Segmental derotation is a technique where axial rotatory forces are applied to individual vertebral segments sequentially to achieve correction. The surgical technique involves placing pedicle screws at appropriate levels, reducing a rod into the screw heads, identifying neutral vertebrae, and applying rotational forces in a stepwise manner while securing each vertebra sequentially. The advantages of segmental derotation include precise control over rotation, maximizing axial derotation forces, and incremental correction to reduce excessive force application. However, it can carry the risk of screw breakout due to concentrated forces while performing the segmental correction.

En bloc derotation involves the simultaneous rotation of multiple vertebrae as a single unit rather than individual segmental correction. This technique begins with pedicle screw placement at all levels, rod positioning, identification of the neutral vertebrae, and the attachment of derotation devices [outriggers], which are interconnected to form a rigid construct. The entire construct is rotated simultaneously while maintaining counterforces at neutral vertebrae and applying a downward force over the rib hump. En bloc derotation distributes corrective forces over multiple vertebrae, reducing stress on individual screws and decreasing the risk of screw pullout. However, it may provide less control over individual vertebrae and result in reduced rotational correction at specific apical levels.

A study analyzing 188 patients undergoing DVBD for AIS found no significant difference in postoperative rib prominence correction between segmental and en bloc techniques. The percentage of curve correction was 63% for segmental, 68% for en bloc, and 64% for the combined technique. Intraoperative analysis revealed that using both techniques led to significantly longer operative duration and higher estimated blood loss [63].

The choice of pedicle screws significantly impacts surgical outcomes when employing derotation techniques. Monoaxial pedicle screws, in contrast to polyaxial screws, have been shown to facilitate superior scoliosis correction and improve rib cage symmetry during DVBD. This advantage stems from the rigid head–body connection, which eliminates rotational freedom at the screw–rod interface. Uniaxial pedicle screws are featuring a pivoting head that moves in only the sagittal plane. This design enables easier rod loading while retaining the derotational benefits of monoaxial screws. In 2011, Wang et al. introduced the Multi-Degree-of-Freedom [MDOF] system, in which screws are linked to rods via post-connectors. This system allows for six degrees of freedom—two translational and four rotational—compared to the two degrees of freedom provided by traditional monoaxial screws. The added flexibility enhances the surgeon’s ability to achieve the desired spinal configuration [64].

In conclusion, the selection of derotation techniques and pedicle screw types should be tailored to individual patient anatomy and deformity characteristics. While segmental derotation allows for greater apical correction and better precision, en bloc derotation provides reduced stress on individual fixation points. Future advancements in instrumentation may further refine these techniques, optimizing outcomes for patients with AIS.

### 5.15. Differential Rod Contouring

Differential rod contouring [DRC] plays a significant role in vertebral derotation and rib hump reduction, supplementing traditional rod rotation methods. The concept of DRC is based on using different contouring angles for the concave and convex rods to generate corrective forces that realign the vertebrae in all three planes—coronal, sagittal, and transverse. Studies have demonstrated that increasing the difference in contouring angles between the two rods significantly improves apical vertebral rotational correction while also affecting the forces exerted on bone–screw connections. Specifically, greater differential rod contouring [with the concave rod bent more than the convex rod] leads to better transverse plane correction but at the cost of increased screw pullout forces and heightened thoracic kyphosis.

Biomechanical studies have shown that increasing the concave rod contouring angle from 35° to 85° results in improved vertebral derotation, increasing from 35% to 68%. However, this also increases thoracic kyphosis from 27% to 144%, which must be considered when planning the correction strategy [65]. Additionally, a study analyzing intraoperative CT scans of AIS patients revealed that DRC contributed significantly to reducing vertebral body rotation, with an average improvement of 6° in apical vertebral rotation following convex rod contouring after concave rod rotation [66]. This finding emphasizes the additive effect of DRC in optimizing three-dimensional spinal correction beyond what can be achieved with rod rotation alone. The effectiveness of DRC also correlates with the degree of curvature difference between the concave and convex rods. It has been observed that when this difference exceeds 10°, vertebral derotation improves substantially, making DRC an essential component of modern scoliosis correction techniques.

Clinically, DRC provides several advantages. First, it allows for controlled and predictable vertebral derotation, improving the alignment of the spine in the axial plane. Second, it reduces the rib hump, an important cosmetic and functional consideration in scoliosis treatment. Third, distributing forces more evenly across the instrumentation potentially enhances the construct’s stability. However, excessive contouring, particularly with highly rigid rods, can increase mechanical stress on the bone–screw interface, leading to potential screw loosening or implant failure. To mitigate this risk, DRC should be carefully planned in conjunction with osteotomies to release the spine and other corrective maneuvers to balance the mechanical forces acting on the spine.

Overall, DRC is a powerful tool in scoliosis surgery, enabling superior vertebral derotation and three-dimensional spinal alignment. By strategically varying rod contouring, surgeons can optimize correction while maintaining biomechanical integrity. Future research should continue refining contouring parameters to maximize correction while minimizing stress on spinal instrumentation that can lead to failure while increasing the neurological risks of the procedure.

### 5.16. Convex Pedicle Screw Technique [67]

**Figure 4:**
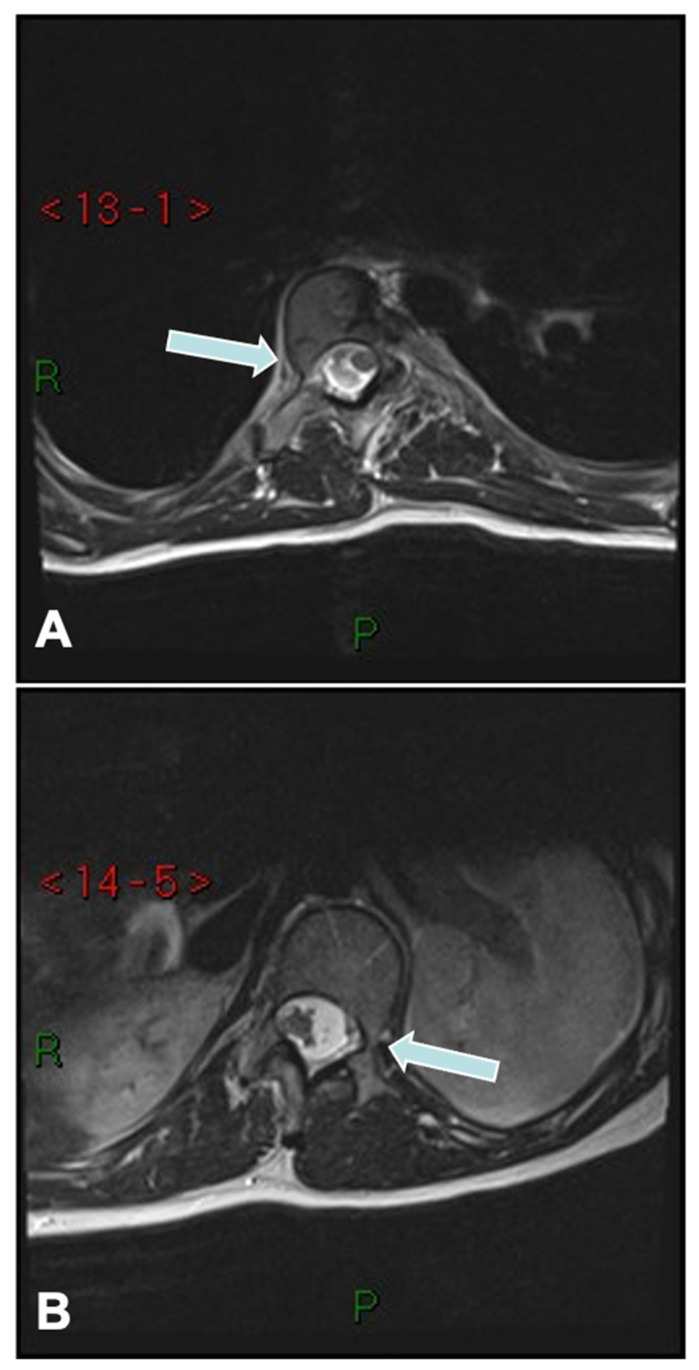
MRI cut sections at (**A**) D8 and (**B**) L1 showing relatively safer convex side pedicles (arrows) with the spinal cord lying at a distance from the convex pedicle when compared to its concave counterpart.

The convex pedicle screw technique in AIS, developed by Tsirikos, is a novel approach that introduced segmental correction through instrumentation and maneuvers applied on the convex side of the spinal deformity. Unlike traditional techniques focusing on concave rod engagement, this method prioritizes convex-side anchoring for improved coronal and sagittal alignment while minimizing neurological and vascular risks. The rationale for using the convex rod technique is based on several advantages, including a lower risk of neurological injury due to the reduced likelihood of medial pedicle breach (Figure 4), as well as the ability to use larger and longer pedicle screws on the convex side, which provide superior purchase and stability. Additionally, this technique facilitates improved coronal plane correction while simultaneously restoring thoracic kyphosis, which is often reduced in AIS. Another key benefit is the use of a lower implant density that reduces surgical time, blood loss, and infection risks while still achieving very satisfactory deformity correction.

The surgical procedure begins with preoperative planning using full-length standing and supine traction scoliosis radiographs complimented by a whole-spine MRI to assess curve flexibility and three-dimensional alignment. This technique can be used for any Lenke type of AIS. The patient is positioned prone on a Jackson table using a Montreal mattress to facilitate spinal alignment and a midline posterior exposure is performed, followed by subperiosteal dissection to the tips of the transverse processes. Facetectomies are routinely performed to increase segmental flexibility, which is essential for optimal correction. Pedicle screws are then placed segmentally on the convex side to allow controlled vertebral translation and coupled derotation, while only two proximal and distal screws are placed on the concave side, in order to augment the construct and provide additional stabilization.

**Figure 5:**
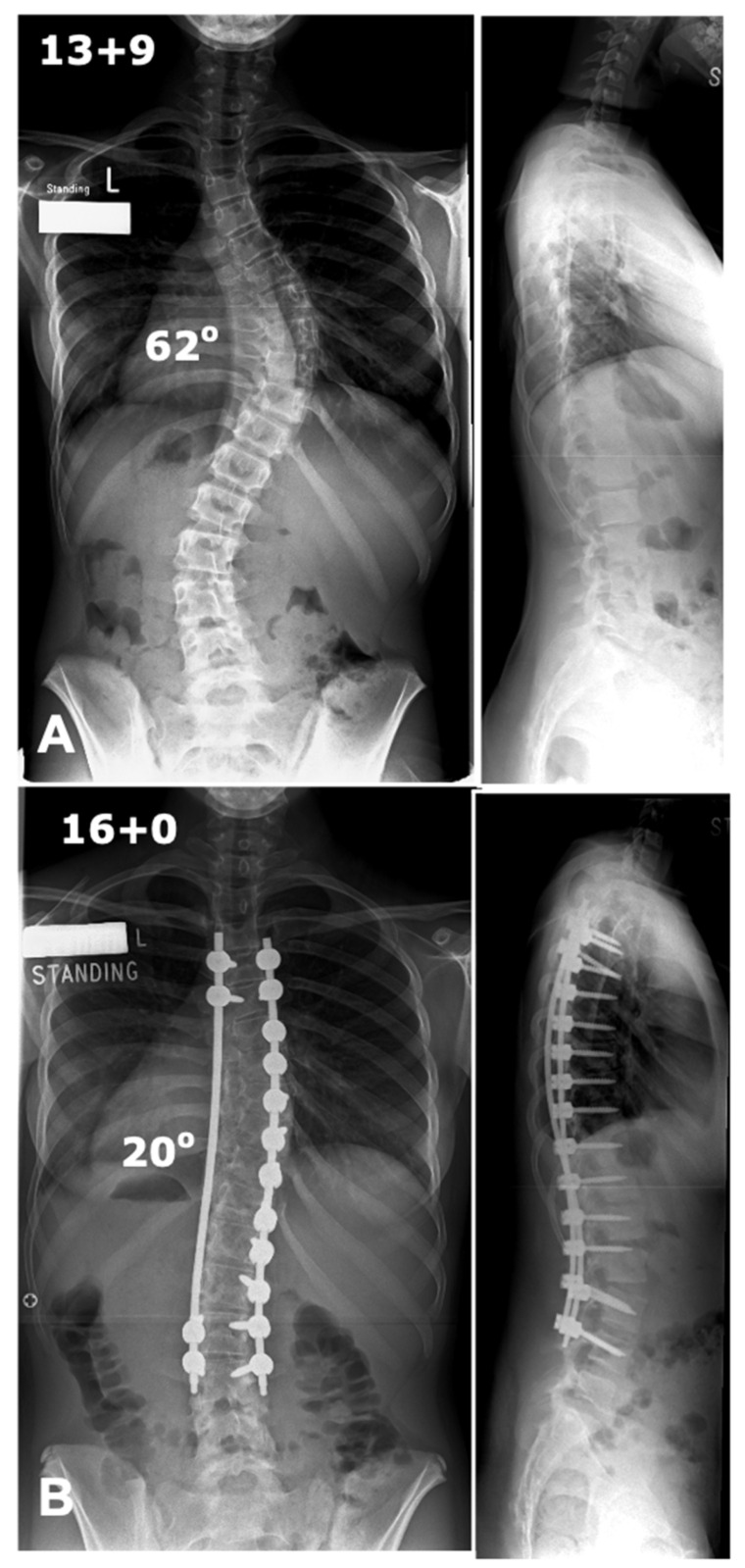
Deformity correction using convex rod technique. (**A**) Preoperative antero-posterior and lateral radiographs showing a thoracic scoliosis with a Cobb angle of 62 degrees. (**B**) Postoperative radiographs showing good deformity correction with coronal and sagittal balance.

The convex rod, which is pre-contoured to restore normal sagittal alignment, is engaged sequentially in the segmental screws. The key initial corrective maneuver involves segmental vertebral translation, where the vertebrae are gradually pulled toward the rod using reduction screws. Direct vertebral derotation is then applied at every level as the convex pedicle screws are locked into the monoaxial screws, further improving the axial deformity. A concave rod is placed once the correction is achieved and the convex rod has been secured to the spine, but its role is primarily supportive rather than corrective. The final step involves the final tightening of the screws on the rods with controlled compression and distraction at the caudal and cephalad ends in order to optimize spinal balance, followed by bone grafting using locally harvested bone and allograft, as needed, to promote fusion. A case example is shown in Figure 5.

Several studies have demonstrated the efficacy of this technique. In a comparative analysis, Tsirikos and Subramanian reported that both bilateral and unilateral screw techniques achieved satisfactory scoliosis correction; however, the unilateral [convex] approach offered reduced surgical time and blood loss, with no significant difference in clinical outcomes including patient-reported results. In addition, Tsirikos et al. applied the convex pedicle screw method across various Lenke curve types, observing significant improvements in patient-reported outcome measures [PROMs] and effective deformity correction [68]. Independent research supports these findings. Ferlic et al. compared a convex pedicle screw technique with low implant density to a traditional bilateral approach, finding comparable correction outcomes with the convex method, alongside shorter operating times and fewer implants [69]. Additionally, a study by Takahashi et al. demonstrated that convex rod rotation maneuvers, combined with direct vertebral rotation, effectively improved vertebral rotation in Lenke types 1 and 2 AIS, confirming this procedure as a viable surgical option [49].

### 5.17. Compression/Distraction

Segmental compression and distraction allow for targeted adjustments of anchor positions along the rod. Distraction induces kyphosis, whereas compression creates lordosis. These techniques are beneficial for correcting alignment, especially in hypokyphotic thoracic curves, but excessive forces must be avoided to prevent implant loosening or failure. Compression/distraction at the proximal end of the construct can level the position of the shoulders, which is often an important aspect of the cosmetic deformity. Equally, application of the same techniques at the distal end of the fusion can make the disc below the instrumentation horizontal, with the aim to achieve adequate alignment of the lumbar spine and reduce the risk of add-on degeneration.

### 5.18. In Situ Rod Contouring

In situ rod contouring is a technique that allows surgeons to modify the rod shape after it has been secured to the spine. This method is particularly useful for correcting rod deflection that may occur following global reduction maneuvers, such as rod derotation or translation. By adjusting the rod’s shape in both the coronal and sagittal planes, in situ contouring helps refine spinal alignment. In cases of thoracic hypokyphosis in AIS, the surgeon can apply a bending force to enhance thoracic kyphosis. However, this technique places significant stress on the bone–implant interface, increasing the risk of implant failure if excessive force is applied. Additionally, in situ contouring is generally less effective with titanium rods due to their elasticity, which causes them to recoil toward their original shape.

## 6. Challenges in Correcting Severe and Rigid Scoliosis

Despite advancements in surgical techniques and instrumentation, managing severe scoliosis [Cobb angle > 70°] or rigid curves [flexibility index < 40%] remains challenging. For extremely rigid deformities exceeding 100°, pre-surgical methods like halo-femoral, halo-tibial, or halo-pelvic traction can be utilized. These methods utilize the viscoelasticity of spinal tissues to gradually reduce deformity over time. However, such techniques are associated with complications and prolonged bed confinement. Halo-gravity traction offers a safer alternative, allowing mobilization in a wheelchair for 2–8 weeks before surgery.

### 6.1. Temporary Internal Distraction

Temporary internal distraction involves the use of distraction rods [similar to a unilateral growing rod construct] to apply controlled forces across a spinal deformity, facilitating correction. These rods may span the entire curve to improve global alignment or target specific regions requiring reduction. This approach is particularly valuable for managing large or rigid spinal deformities and can serve as an alternative to halo-gravity traction, especially for patients with cervical pathology that precludes cranial traction [70]. Temporary distraction is often combined with osteotomies and soft tissue releases, either in a single-stage surgery or as part of a staged correction. This technique carries a significant risk of intraoperative neuromonitoring changes, with reported rates as high as 41%, although most of these changes are reversible. Additionally, the use of temporary distraction rods increases the risk of anchor failure, and often these anchors cannot be used as permanent fixation points at the upper or lower instrumented vertebrae [71,72].

A specific application of temporary internal distraction is in the treatment of Lenke type 2 [double thoracic] curves, where a temporary rod is used to reduce the structural proximal thoracic curve. By applying traction between the upper and lower end vertebrae of the proximal thoracic segment, the characteristic sigmoid deformity is converted into a single thoracic curve, making it more amenable to other corrective maneuvers, such as rod derotation. This technique, first described by Sudo et al., demonstrated effective correction with a significant increase in thoracic kyphosis from an average of 9.3° preoperatively to 19° at final follow-up, with all patients achieving either balanced or mildly unbalanced shoulders [73].

### 6.2. Halo Traction

Halo traction can have a role in the management of severe AIS, particularly in patients with very rigid deformities. As a preoperative adjunct, it provides gradual, controlled correction of the spinal curvature while reducing the risk of neurological complications that can occur with an acute curve correction. Halo-gravity traction [HGT], in particular, has been widely utilized for its ability to improve spinal flexibility, enhance pulmonary function, and facilitate safer surgical correction [74]. The technique involves the application of a halo ring secured to the skull, which is progressively loaded with weights to apply longitudinal traction along the spine. This gradual stretching allows for soft tissue adaptation, vertebral realignment, and improved correction potential, reducing the force required during definitive spinal fusion. Studies have demonstrated that HGT significantly reduces the coronal Cobb angle and sagittal plane deformities, with reported improvements in forced vital capacity [FVC] and forced expiratory volume [FEV1], which are critical in patients with compromised pulmonary function [74,75]. Meta-analyses have confirmed that HGT achieves a mean reduction of approximately 27° in the coronal Cobb angle before surgery, further increasing correction efficiency when followed by spinal fusion. Additionally, it has been found to contribute to better nutritional status in patients, as improved spinal alignment facilitates better gastrointestinal function [75]. Compared to other forms of traction, such as halo-pelvic traction, HGT allows for patient mobility, reducing the risks associated with prolonged bed rest, including respiratory infections and pressure sores. While effective, the technique is not without risks; complications such as pin site infections, cranial nerve palsies, and traction-related discomfort have been reported, but are generally manageable with careful monitoring [76]. In a three-stage correction protocol, where HGT is combined with anterior release followed by posterior instrumentation, better overall curve correction and shorter operative times have been achieved compared to traditional two-stage approaches [77]. Ultimately, halo traction serves as a useful tool in the surgical treatment of severe AIS, offering a means to enhance correction, improve physiological parameters, and reduce intraoperative risks, making complex spinal deformity surgeries safer and more effective.

## 7. Anterior Versus Posterior Fusion in Lenke Type 5 Curves

**Figure 6:**
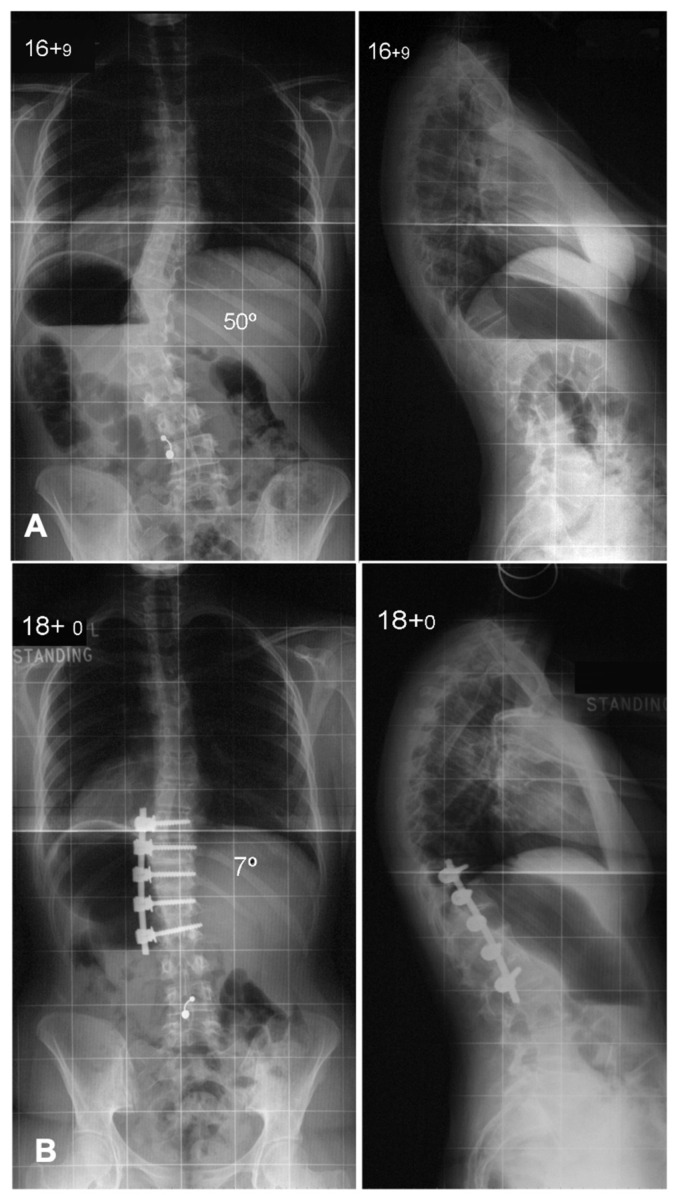
Deformity correction using anterior spinal fusion in a Lenke 5 curve. (**A**) Preoperative antero-posterior and lateral radiographs showing a thoraco-lumbar scoliosis with a Cobb angle of 50 degrees. (**B**) Postoperative radiographs showing good deformity correction with coronal and sagittal balance. Anterior spinal fusion allows good deformity correction with shorter fusion constructs.

**Figure 7:**
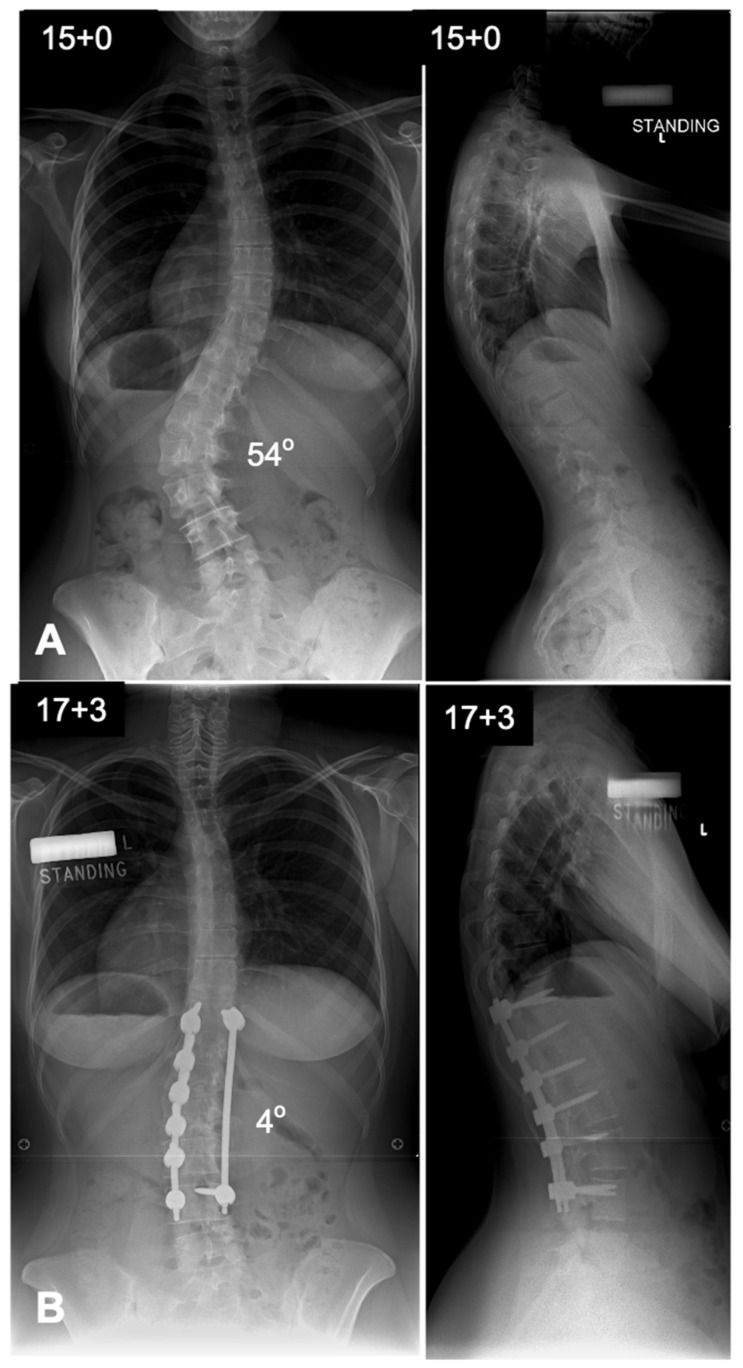
Deformity correction using posterior spinal fusion in a Lenke 5 curve. (**A**) Preoperative antero-posterior and lateral radiographs showing a thoraco-lumbar scoliosis with a Cobb angle of 54 degrees. (**B**) Postoperative radiographs showing good deformity correction with coronal and sagittal balance. Correction in posterior spinal fusion in thoraco-lumbar and lumbar curves is achieved from the convex rod.

Lenke 5 AIS, characterized by a primary thoracolumbar/lumbar [TL/L] curve with a compensatory thoracic curve, can be managed with either anterior spinal fusion [ASF] (Figure 6) or posterior spinal fusion [PSF] (Figure 7). The key argument favoring ASF is that it allows for shorter fusion constructs, potentially preserving an additional distal motion segment, which may reduce the risk of low back pain in later life if the fusion can be stopped at L3 rather than L4 [16]. Several studies have compared these approaches, showing that ASF achieves comparable coronal correction with fewer fused segments [17]. However, PSF, facilitated by modern segmental pedicle screw constructs, has become the standard approach due to its technical familiarity and ability to provide superior three-dimensional correction, particularly in terms of lumbar lordosis and thoracic kyphosis restoration [11,18]. Previous studies have indicated a greater loss of sagittal plane correction over time after ASF compared to PSF [17]. Additionally, PSF has been associated with better compensatory thoracic curve correction and improved trunk shift realignment [16,17]. Importantly, factors such as curve magnitude, lowest instrumented vertebra angle, and apical vertebra translation influence the decision on how far to extend the fusion distally. A predictive equation has been developed to guide this decision in PSF, helping to determine whether stopping at the Cobb-to-Cobb levels of the curve is sufficient or if additional distal levels should be included, in which case there may be an indication to consider performing an ASF instead [18]. Ultimately, both approaches have their strengths, and the choice should be individualized based on patient anatomy, surgical goals, and the surgeon’s expertise.

## 8. Rib Hump Deformity

**Figure 8:**
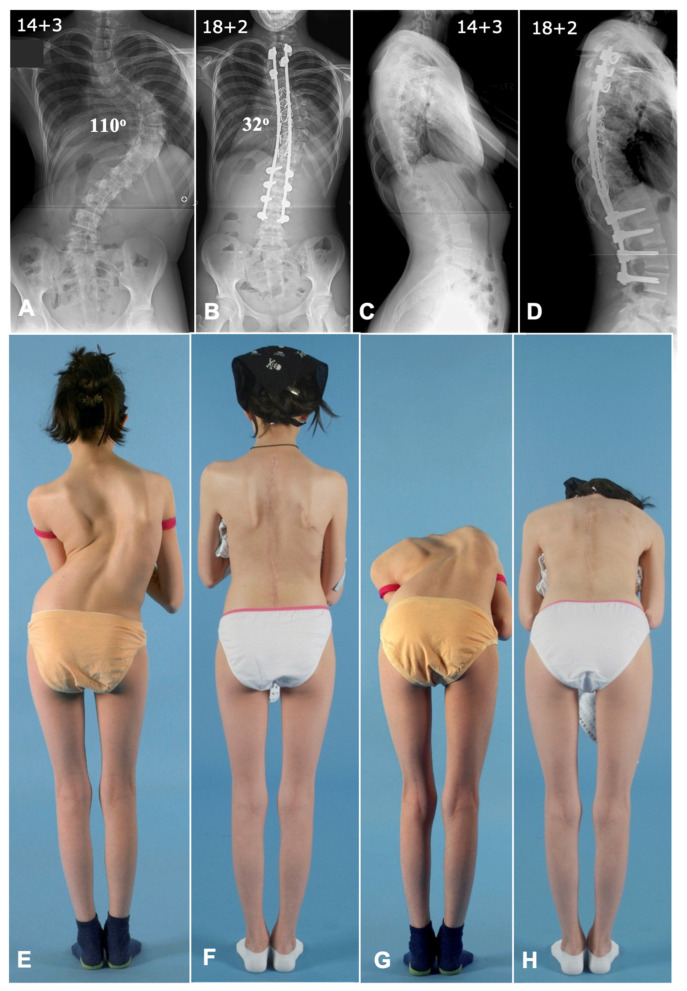
Deformity correction using a combined anterior and posterior approach in a severe rigid thoracic scoliosis case. (**A**,**C**) Preoperative antero-posterior and lateral radiographs showing a thoracic scoliosis with a Cobb angle of 110 degrees. (**B**,**D**) Postoperative radiographs showing good deformity correction with a Cobb angle of 32 degrees with well-maintained coronal and sagittal balance.(**E**,**G**) Preoperative clinical photographs showing a severe deformity and rib hump. (**F**,**H**) Postoperative clinical photographs showing a good correction of deformity and a well-maintained balance. Note the correction of the convex rib hump by anterior thoracoplasty.

One of the most cosmetically significant aspects of scoliosis is the rib hump deformity associated with the axial rotation of the scoliotic curve. This deformity significantly impacts patient satisfaction post-surgery. Thoracoplasty, which involves removing pieces from several consecutive ribs, can enhance thoracic flexibility [if this is performed on the concave side] and improve rib hump correction [if this is performed on the convex side]. The excised ribs can also serve as bone grafts for fusion. Convex anterior thoracoplasty, as part of releasing a stiff thoracic scoliosis, produces a more predictable reduction in the rib prominence when compared to posterior thoracoplasty as it allows the removal of the rib heads across multiple levels that consequently reduces the sharp gibus of the chest wall on the deforming side of the curve (Figure 8).

Correction techniques such as simple rod derotation [SRD] and direct vertebral body derotation [DVBD] utilize three-column vertebral control, employing segmental or en bloc maneuvers. Thoracoplasty can be effective in reducing the risk of recurrence of rib prominence and apical vertebral rotation in severe cases. However, in mild deformities, DVBD alone may achieve comparable results.

## 9. MISS Versus Open Surgery in AIS

Minimally invasive scoliosis surgery [MISS] has emerged as an alternative to conventional open scoliosis surgery [COSS] for the correction of AIS. Traditional open surgery has been the gold standard for achieving spinal deformity correction through an extensive exposure, pedicle screw fixation, and rod derotation maneuvers. However, this approach is associated with significant soft tissue disruption, increased blood loss, longer hospital stays, and larger surgical scars. The advent of MISS aims to minimize surgical trauma by using smaller incisions, muscle-sparing techniques, and intraoperative navigation, which theoretically leads to reduced perioperative morbidity and improved cosmetic outcomes. Comparative analysis of MISS and open surgery in AIS has shown that both techniques achieve similar curve correction. Studies indicate that the correction rate for MISS is approximately 73.2%, while open surgery achieves 76.7%, with no significant difference in postoperative coronal or sagittal balance [78,79]. MISS has been associated with significantly lower blood loss, with reports showing an average estimated blood loss [EBL] of 271 mL in the MISS group compared to 527 mL in the open surgery group. This reduction in blood loss is a major advantage, as it can decrease the need for transfusions and associated risks. Despite this, operative time is consistently longer in MISS, with an average of 380 min, compared to 302 min for open surgery, likely due to the technical challenges of performing spinal deformity correction through limited exposure [78].

In terms of patient recovery, MISS has demonstrated advantages in reducing postoperative pain and hospital stay. Some studies report that patients undergoing MISS have a shorter hospital stay [5.1 days] compared to open surgery [6.4 days], likely due to less soft tissue damage and a faster return to mobility. Additionally, MISS is associated with smaller surgical scars, which may be particularly relevant for adolescent patients concerned about cosmesis [78]. However, complications such as rod dislodgement, wound infections, and hypertrophic scarring have been reported in early cases of MISS, highlighting the need for careful patient selection and surgical expertise [80].

Despite these benefits, MISS has certain limitations. The technique is more technically demanding, requiring specialized training and experience. Additionally, some studies suggest that MISS may be less effective in correcting large or rigid curves, making it less suitable for severe scoliosis cases. While MISS offers advantages such as reduced blood loss, shorter recovery time, and better cosmetic results, open surgery remains the preferred approach for achieving maximum correction in complex deformities while increasing the ability to secure a fusion across the operated levels due to adequate bone grafting. Future research is needed to further refine MISS techniques and improve outcomes to match the effectiveness of traditional open surgery while minimizing its drawbacks.

## 10. Complications

Surgical management of adolescent idiopathic scoliosis [AIS] is generally safe and effective but complications can arise despite advances in surgical techniques and instrumentation. Neurological complications, while rare, remain among the most feared, with reported incidences ranging from 0.3% to 4% [81]. These can range from transient neuropraxia due to positioning to severe spinal cord injuries leading to irreversible paralysis. Intraoperative hypotension, excessive spinal cord traction, and misplaced implants are key risk factors. Multimodal intraoperative neuromonitoring plays a crucial role in detecting and preventing such injuries, with studies showing high sensitivity and specificity for detecting early neurological changes [82]. Tsirikos et al. developed an algorithm for responding to motor-evoked potential [MEP] loss, which has been adopted by other spinal units across the UK, improving intraoperative decision-making and patient safety [83].

Infection and wound complications are another a significant concern in AIS surgery, with surgical site infections [SSIs] reported to occur in 0.17% to 9% of cases. Risk factors include prolonged operative time, extensive blood loss, and the presence of non-idiopathic scoliosis. Most infections occur in a delayed fashion, often months after surgery, and are predominantly caused by skin flora such as Staphylococcus aureus. Management typically involves irrigation, debridement, and targeted antibiotic therapy, with implant retention attempted whenever possible. Negative pressure wound therapy has been shown to improve outcomes in cases of deep infection with poor soft tissue cover. Efforts to reduce infection rates include perioperative antibiotic prophylaxis, meticulous surgical techniques that can reduce soft tissue damage, and strict aseptic protocols.

Implant-related complications, including instrumentation failure, rod fractures, and screw loosening, occur in approximately 0.64% to 1.37% of cases. Factors contributing to these issues include inadequate fixation, poor bone quality, and excessive mechanical stress. Additionally, complications such as proximal junctional kyphosis [PJK] and the crankshaft phenomenon remain concerns, particularly in younger patients with significant growth remaining. Strategies to mitigate these risks include careful preoperative planning, appropriate implant selection, and avoiding the overcorrection of deformities. Despite these challenges, the overall complication rates in AIS surgery have declined in recent years, reflecting a better understanding of deformity considerations, as well as improvements in surgical techniques, perioperative care, and patient outcomes.

## 11. Conclusions

In conclusion, the evolution of surgical techniques for AIS has significantly improved correction outcomes, patient safety, and long-term spinal balance. Advancements in instrumentation, correction maneuvers, and preoperative planning have allowed for precise three-dimensional deformity correction while reducing the risk of complications. However, despite technical progress, assessing surgical success should not solely rely on radiographic and intraoperative parameters. Patient-reported outcome measures [PROMs] provide an objective and patient-centered evaluation of surgical efficacy, functional recovery, and quality of life. The study by Tsirikos and Garcia-Martinez highlights the importance of long-term health-related quality of life assessments in pediatric spinal deformity surgery, showing that outcomes must be compared with the general population to fully understand the impact of interventions [84]. Future research should integrate PROMs into AIS surgical studies to refine treatment strategies and enhance evidence-based patient care.

## Figures and Tables

**Table 1:** Lenke classification for adolescent idiopathic scoliosis [7].

Lenke Type	Curve Pattern	Major Curve Region	Structural Secondary Curves	Common Characteristics
Type 1	Main Thoracic (MT)	Thoracic	Non-structural proximal thoracic (PT) and lumbar (L)	Most common, “C” shaped
Type 2	Double Thoracic (DT)	Thoracic	Structural PT, non-structural L	Structural PT curve differentiates from Type 1
Type 3	Double Major (DM)	Thoracic and lumbar	Both MT and L structural	Nearly equal MT and L curves
Type 4	Triple Major (TM)	Thoracic, lumbar, and PT	All three structural	Balanced three-curve pattern
Type 5	Thoracolumbar/Lumbar (TL/L)	Thoracolumbar or lumbar	Non-structural MT	Primary curve in TL or L region
Type 6	Thoracolumbar/Lumbar—Main Thoracic (TL/L-MT)	Thoracolumbar or lumbar	Structural MT	TL/L curve larger but MT is structural
Lumbar Modifier (Based on Center Sacral Vertical Line—CSVL):				
A: CSVL passes between lumbar apex pedicles (neutral)				
B: CSVL touches the concave pedicle (intermediate)				
C: CSVL does not touch the apex pedicle (coronal imbalance)				
Sagittal Modifier (T5–T12 Sagittal Profile):				
−: Hypokyphosis (<10°)				
N: Normal (10–40°)				
+: Hyperkyphosis (>40°)				

**Table 2:** This table lists studies employing different techniques in the surgical management of AIS.

Study [Year]	Type	N	Lenke Type			Correction Maneuver	Coronal CR	AVR CR	TK CR
Delorme et al. (1999) [42]	Prospective	70	King Type 1–5			Group 1 [RR]: 39; Group 2 [T]: 31	62.1%	-	-
Lee et al. (2004) [43]	Prospective	38				DVR [17] RD [21]	Thoracic: DVR [79.6%] RD [68.9%] Lumbar: DVR [80.5%] RD [62.2%]	42.5% [DVR]; 2.4% [RD]	-
Tsirikos et al. (2012) [44]	Retrospective	Bilateral Rod: 51		Group 1	Group 2	VT and DVR	PT: 71% vs. 55% MT 71% vs. 70% TL/L: 74% vs. 70%	-	-
		Unilateral Rod: 161	Type 1:	15	44				
			Type 2:	4	12				
			Type 3:	18	49				
			Type 4:	5	9				
			Type 5:	3	32				
			Type 6:	6	15				
Sudo et al. (2014) [45]	Retrospective	32	Type 1			SDRR	67.8%	24.36%	41.95%
Pankowski et al. (2016) [46]	Prospective	38	Type 1 [26]; Type 3 [4], Type 5 [8]			SCRR and DVR	64% for thoracic curve and 80% for lumbar curve	SCRR: 16.1% worsening DVR: 34.4%	-
Ohrt-Nissen et al. (2017) [47]	Retrospective	H/H: 65; APS: 64		H/H	APS		H/H: 31%; APS: 49%	-	Better in H/H group
			Type 1:	46	30				
			Type 2:	24	10				
			Type 3:	4	4				
			Type 4:	2	7				
			Type 5:	2	8				
			Type 6:	7	5				
Violas et al. (2019) [48]	Prospective	23	Type 1: 5; Type 2: 15 and Type 3: 3			Convex rod rotation	45.83%	42.3%	23.8%
Takahashi et al (2021) [49]	Prospective	59	Type 1 and 2			CRR+DVR	Lenke type 1: 75.1%; Lenke Type 2: 65%	-	-
Pesenti et al. (2022) [50]	Retrospective comparative study	562	Lenke Type 1			ISB, RD, cantilever, and PMT	ISB 64% vs. C 57% vs. RD 55% vs. PMT 67%	-	PMT group [average + 13°], DR [+3°], ISB [−3°], and C [−13°]

## Data Availability

Data can be made available on request.
